# Dental Hints Department

**Published:** 1906-08

**Authors:** B. H. Teague

**Affiliations:** Aiken, S. C.


### DENTAL HINTS DEPARTMENT

**Conducted by B. H. Teague, D. D. S., Aiken, S. C., to whom all contributions to this department should be sent.**

Ink and fruit stains may be removed from white linens and cottons by soaking them for a few hours in kerosene, then washing in hot water.

Dentistry may be considered as twin brother to surgery and the relation was early recognized in the naming of our specialty "dental surgery."—S. H. Guilford, *Stom.*

He who would extract a tooth knowing that a brother dentist had refused ethically to do so, is a — — well, a Judas.

The oxidizing agent that has proven most servicable to the dentist is the familiar peroxide of hydrogen ($H^2O^2$) or Aqua hydrogenii dioxidi, U. S. P.—Henry H. Boom, *Stom.*

Society Sends $700 to Frisco Dentists.—At the session of the Dental Society of the state of New York in May, $700 was raised for the relief of the San Francisco sufferers.—*Summary.*

Presentation of a Loving Cup.—The dentists of Florida at their state convention presented Dr. J. S. W. Gallagher of Winona, Minn., with a handsome loving cup in order to show their appreciation of a clinic given by him at the convention.—*Summary.*

The dental profession is too much interested in "doing things" at the present time to bother about the "theories and speculations of the scientific why's and wherefores."—*Critic.*

The members of the Georgia Dental Society, presented Dr. H. A. Lowrance, their retiring treasurer, with a handsome testimonial of their love and regard. Dr. Lowrance had been reelected to office for many years.

A baking powder-can with a ragged hole punched in the top and covered with colored paper. pasted on, makes a good receptacle for discarded pellets, rolls, etc.

When you extract a tooth you have to see that you encourage bleeding after a local anesthetic. Make the gums bleed well and use a strong saline solution and you will not have any trouble in the use of the local anesthetic.—Dr. J. K. Conroy, *Era*.

There should be a dentist on every hospital staff, on the medical staff of every home for the orphan or the aged and of every other public or eleemosynary institution. I will go further and state that every institution that requires a physician should also have a dentist.—Dr. F. W. Wiseman, *Era*.

To such dental practitioners, as contemplate adding an oxidizing agent to a dentifrice composition, our advice is that of "Josh Bilings" to the poor young man contemplating matrimony—"Don't."—Henry H. Boom, *Stom'*.

Such faith have I in the efficacy of preventive measures that I do not hesitate to declare that the child is already born who, barring accident or systemic disease, will never know the meaning of dental discomfort.—S. H. Guilford, *Stom'*.

To-day the chief glory of dentistry lies, not in the sacrifice of important organs but in their conservation, thus adding both to the health and comfort of the human race. Experiment, investigation and tentative practice are exhibiting results in the way of prevention scarcely dreamed of even by our immediate predecessors.—S. H. Guilford, *Stom'*.

Charcoal as an Antidote to Mushroom Poisoning.—The *Bulletin general de therapeutique*, April 15, 1906 states that pulverized wood charcoal, or preferably animal charcoal, is an efficient antidote to the poison of mushrooms, acting almost miraculously. Several spoonfuls of animal charcoal mixed with water are sufficient to relieve or check the most severe cases of poisoning.—*Stom'*.

Puncture in Dam.—For punctures of the dam or to stop leakage around the lower anterior teeth, dry the dam with cotton, melt a little baseplate wax on a spatula and carry to points of leakage. This result will gratify you.—Dr. A. C. Peterson, *Tri-State Quarterly*.

Cementing Arsenic.—Mix the cement rather thin and place a small drop on a small bit of paper and carry the paper to the cavity with the pliers. Press the place with a burnisher. The paper facilitates adjustment to place and prevents cement adhering to instrument.—Dr. C. B. Warner *Tri-State Quarterly*.

Code of Ethics.—Let there be a code of ethics that we may all understand, and then let all who are unwilling to act together in harmony, for the interest of our patients and the profession, and for themselves as well, be relegated to the rear and be counted outside the pale of respectability.—C. W. HUNTINGTON,*Stom'*.

TO PROTECT UPPERTEETH FROM SALIVA.—Place cotton roll on buccal side, then cover lower teeth and tongue with a square of rubber dam 3 by 2 inches, and confine the latter to place with a clamp pressed over the dam onto a lower molar tooth. If saliva flows freely use ejector also.

FLOW OF SOLDER.—In soldering gold, when it is desired to restrict the flow of solder to certain areas, with a sharp lead pencil draw a line around the desired area; the solder will not flow past the line. This is especialy valuable in filling cusps of seamless crowns, to prevent the solder flowing up the sides of the crown.—DR. F. W. FRANKLIN, *Western Dental Journal.*

At the meeting of the American Dental Association in Cincinnati in 1867 I offered this resolution: "That this association offer a prize of $5,000 to any dentist, chemist or other person who may invent a *plastic* substance for filling the teeth which shall answer all the purposes of gold, but the color of which shall harmonize with that of the teeth." Adjournment came on and the subject was not called up again.—DR. A. W. FRENCH, *Era.*

The National Dental Critic is sent to DENTISTS, and if "decency and good order are ever to be realized in the National Dental Association, DENTISTS must be given knowledge as to the present state of affairs, WHICH THAT EDITOR WOULD COVER UP by private compromise behind closed doors for fear of the effect of the knowledge upon the minds of the decent and honest element of the profession. We are not afraid of any dishonor coming to the profession through the exposing of its vices, or loss of respect from the public by something IT DOES NOT SEE.—*Critic.*

TEMPERING GOLD.—Z. F. Vaughn,of Los Angeles,Cal., is reported to have discovered a process of tempering gold and other metals.

By his method of tempering it is claimed that the ductile metals are not only hardened, but their density and homogeneity are brought to such practical perfection that a cutting edge is given keener and more durable than steel, because of the microscopic fineness and smoothness impartd to it.—*Stom'.*

HYDROGEN PEROXIDE IN SKIN COSMETICS.—H. Keuhl states that glycerin and rose water used in the treatment of chapped hands is improved by the addition of hydrogen peroxide, thus: Glycerin, 2; rose water, 2; hydrogen peroxide, 1. Mix. It may also be mixed with ointments for the treatment of skin affections. Intimately mixed with lanum, it produces a smooth, soft, white cream, which may be used to bleach the skin. A mixture of equal parts of lanum and zinc ointment, combined with hydrogen peroxide also forms a useful ointment for the same purpose.—*Stom'.*

Senistive Cavities.—When a cavity is very sensitive I usually precede with cement and allow it to remain 60 or 90 days, and then fill over the cement by removing a small film of the cement, but the most difficulty I encounter in the shallow cavities is that I cannot go deep enough to make the combination filing; where it can be done we have a good substantial filing with gold. Many operators deming the insertion of gold fillings unwarrented under the conditions I have named.—Dr. W. G. Hamm, *Summary.*

Erosion.—Erosion is of two kinds—is confined to two descriptions: erosion proper, so-called and abrasion. Lactic acid is generally the active agent in producing it. After the article published by Dr. W. D. Miller, recording the use of silver nitrate, as a prophylactic, we have the use of paraffine as a suggestion for arresting caries, as well as for retarding erosion. Melted paraffine is an excellent agent; I regard it as an efficient agent in treating erosion, but do not believe it is to be depended on to arrest caries. I was surprised to learn from Dr Miller's paper that silver nitrate is not efficient in arresting erosion.—Dr. .H T. Smith, *Summary.*

Where Pressure Anesthesia Fails.—Teeth of old persons; teeth of inveterate tobacco chewers; worn, abraded and eroded teeth; teeth with extensive secondary calcific deposits; teeth whose pulp canals are obstructed by pulp nodules; teeth with metallic oxides in tubules; teeth with leaky old fillings; badly calcified teeth—mainly all from one and the same cause, namely, clogged tubuli. In most of such cases no amount of persistent pressure will prove successful.—Geo. Zederbaum, *Dental Register.*

Shrinkage in Rubber During Vulcanizing.—Dr. G. B. Snow says the amount of shrinkage depends not alone on the time the rubber is subjected to the process of vulcanization, but also upon the temperature. The lower the temperature and steam pressure, the less the loss in shrinkage and the less the contraction in cooling. Low heat and long time also insure an improvement in the texture of the product.—*Digest.*

Make Your Artificial Dentures Yourselves.—It is difficult to see how satisfactory results are obtained by those practitioners who, in the parlance of Christian Science, apply "absent treatment," by taking the impression and bite, themselves, and then have a laboratory man who has never met the patient do the remainder of the work.—Charles R. Turner, *Review.*

Antral Abscess Diagnosis.—If an antral abscess is suspected, let the patient blow his nose thoroughly upon the affected side until no pus is expelled. Then let him, with feet together, endeavor to touch his toes without bending the knees, for from one to two minutes. Extensive aching of the diseased antrum and eventually of the diseased teeth will be induced and a stream of pus will result from renewed blowing of the nose.—H. Stolte, *Cosmos.*

I have a suggestion to offer regarding annealing. In offices in which the alcohol flame is to be used, if gold is kept in ammonia fumes, and thus kept from contamination with other gases (an ammonia salt is formed, and that ammonia salt is the most volatile of all common salts), the gold is more easily annealed. The salts or gases that are on the so-called non-cohesive golds are not as easily driven off.—Dr. Sprague, *Digest.*

Fatalities.—May 4, a Canadian succummed at Los Angeles, Cal., to blood poisoning from a dental operation.—May 12, a young man died from cirrhosis of the jaw at the University of Pennsylvania Dental College, resulting from an ulcerated tooth. —May 17, a noted criminal lawyer of Knoxville, Tenn., died from blood poisoning, the result of an ulcerated tooth.—May 19, a 7-year-old boy of Belleville, Mo., died of blood poisoning, following the removal of an ulcerated tooth.—May 21, an Indianapolis merchant succumbed to blood poisoning, which followed the extraction of a diseased tooth.—*Digest.*

In the use of strong solution of nitrate of silver it is necessary to neutralize any of the solution which escapes from the confines of the area intended for its reception for nitrate of silver solutions, except the very weak ones, are escharotic.

For that purpose I am in the habit of flushing out the mouth with a solution of common salt, say 50 per cent, but most any strength will do. In that way the insoluble silver chloride is formed.

Silver chloride when dry has a sandy consistency, so that it should be well washed out of a cavity. DR. O. HOLINGER, *Review.*

Not long since I was asked to locate the cause of a persistent pain in a lower cuspid in which the pulp had been removed and canal filled. The filling had been carefully removed, so that I might see that the canal had been opened to the apex of the root. I enlarged the cavity in the lingual surface of the tooth which was altogether too small for thorough cleansing, or to allow sufficient space for exploration. I found that the tooth had a supernumerary root, in which the pulp was vital—hence the pain. The pulp was removed and the trouble ceased. From the fact that the pain was the same as that in pulpitis, I was able to make the diagnosis.—DR. E. CURTIS, *Items.*

CAVITY MARGINS FOR INLAYS.—Every portion of the cavity, and in particular the edges, should not only be well shaped, but brilliantly polished. With small Arkansas stone points a beautiful finish can be obtained, which should extend over the edges, giving everywhere in the neighborhood of the cavity a polished surface from which it is easy to remove the matrix. It is folly to attempt making a perfect matrix against a sharp, uneven, or rough edge; or to expect always to remove it without accident from a cavity around which rough surfaces have been left.—N. S. JENKINS, *Elliott's Quarterly.*

FORMULA FOR LIQUID FLUX.—The best liquid flux that I have ever used, which I compound myself and find ever ready and faithful, is—

℞—Boric acid,

Borax, aa ℥ss;

Ammoniæ muriat., gr. xij;

Pot. carb. gr. v;

Aquæ dest. q. s. ad ℥iv. M.

It will keep indefinitely and can be used on any metal for hard soldering. It should not be used, however, in connection with porcelain.—H. E. DAVIS, *Dental Era.*

WASTE.—At a meeting of a near-by local society Dr. F. M. Rood, in a side discussion of this very subject, said he had recently sent to a refiner four ounces of polishing strips and disks, for which he received two dollars and forty cents; had he offered the strips to any one of the majority of those present at ten cents a pound it is a question if the offer would have been accepted. And yet the poor men of the profession are throwing away annually strips, disks, filings, clippings, grindings, etc., by the thousands of pounds, a suggestion of which, if done by an employee in a manufacturing jeweler's establishment would lead to manslaughter in the first degree. And yet we go on wondering why we have to wear last year's hat, shoes run down at the heel, and trousers bewhiskered at the ends of the legs!—*Dental Office and Laboratory.*

Dish for Acid Bottles.—Set your laboratory acid bottles in a glass dish, or granite or other glazed tray, first covering the bottom with a thin layer of washing soda. The last-mentioned article takes up the drip from the neck of the bottles, also to some degree the heavy fumes. If it is desired to have a dish to each bottle, the sponge dishes in use by bank cashiers and dentists, generally, when counting their money, answer the purpose perfectly.—*Dental Office and Laboratory.*

We are aware that it is claimed "that every man in the South has been pledged" to do certain acts at Atlanta next fall, but we do not believe the assertion for the following reasons:

In the first place the South is made up of 96 per cent American born citizens, and Americans love FAIR PLAY, HONESTY AND DECENCY, and notwithstanding the political clique have strong and forcible followers in that section whose influence in politics carry great weight, we do not believe they can carry HONEST AMERICANS INTO DISHONEST TRANSACTIONS, and we think it quite presumptuous of the clique to claim they will do so. We will patiently await results before passing judgment.—*Critic.*

The man who so far forgets the dignity of so honorable a profession as dentistry as to quote prices for this, that or the other kind of work, has a degraded opinion of his profession and confessedly no kindred feeling with his fellow practitioners. In the practice of our profession we are entitled to a fair remuneration for our work; for instance the restoration of a broken-down tooth with a beautiful and useful gold or porcelain crown is worth, even though the actual cost to the operator may not be in cash more than 50 cents to $2, not less than $8, and from that on up to $10 or $15. To produce such a piece of work requires skill and patience; no one can do it but a dentist, and if dentist would not do it for less our patients would gladly pay the price and appreciate the work far more than at the ridiculous price of $3 or $5 or $6.—C. W. Huntington, *Stom.*

Selecting a Crown.—In matching all porcelain crowns the shade can be determined more accurately by examining both crown and natural teeth from the lingual aspect wtih the mouth mirror, while holding the crown in position in the mouth. In some cases a crown may seem to be a perfect match while looking at it from the labial side only, when, upon examination from the lingual side, there will be disclosed a distinct difference in shade. A crown should be selected having for its foundation body material of the same shade as the underlying colors in all angles of light, but will match more closely after a few years of service, when the natural teeth gradually assume a darker shade.—P. P. DORR, D. D. S., *Dental Brief.*

THE ANTISEPTIC VALUE OF CHLORETONE.—Four or five years' use of chloretone has demonstrated the practical utility of an alcoholic solution for topical application previous to the use of the hypodermic needle in the gums. A ten or twenty per cent solution in seventy-five per cent alcohol may be used, and unlike cocain causes no toxic effect. An application for this purpose should have the following qualities: First, detergent; second, antiseptic; third, anesthetic. The alcohol posesses the first requirement, cutting the mucus and leaving the membrane absolutely clean. The tissue is then readily acted upon by the antiseptic agents, alcohol and chloretone, and sterilization of the field of operation results. Finally, the anesthetic action of the chloretone is had on the tissues and the hypodermic needle can be used with a minimum of pain and no possibility of septic infection.—DR. T. A. GORMLEY, *Register.*

TREATING AN ALVEOLAR ABSCESS.—I believe it is a mistake to carry a tooth along through weeks and weeks of treatment through the pulp chamber, and many of us make a mistake in treating teeth too often. Whenever an abscess forms about a root it nearly always surrounds the apex of the root. It destroys the peridental membrane around the sides of the root, consequently treatment through the root canal does not get at what we want, and after pus has been present for some time, if the surface of the root has been bathed in pus for many weeks, we will get satisfactory results from treatment through the pulp chamber in very few cases. I do not practice immediate root filling in such cases, but I have done it "more immediately" than is the general custom. In such cases I prefer to fill the root canal, and, if necessary, make an opening through the alveolar process.—ARTHUR D. BLACK, *Review.*

DEEP INJECTIONS OF ALCOHOL IN NEURALGIA.—Ostwalt states that he has made 250 deep injections of alcohol in cases of tic douloureux or facial neuralgia, and never has had the slightest mishap or unpleasant by-effect. The pain was arrested at once. The effect is like a transient Gasserectomy, as for the time being the functions of the Gasserian ganglion are suspended. In at least 90 per cent of the cases the neuralgia was cured by the procedure. In about a third of the patients, recurrence was observed after four or five months, but one or two more injections definitely banished the pain. He injects 1 or 1.5 c.c. of 80 per cent alcohol, to which .01 gm. of cocain or stovain has been added, making the injection along the trunk of each of the branches affected at the point where they emerge from the bone. He prefers a bayonet-shaped needle and has found these deep injections of alcohol effectual in case of neuralgia elsewhere in the body, in sciatica, etc. He has also cured cases of rebellious facial hemi-spasm by injecting, a drop at a time, 70 per cent alcohol along the trunk of the facial nerve, according to Schlösser's technic.—*Journal American Medical Association.*

Thymophen.—Equal parts by weight of crystallized carbolic acid and thymol crystals rubbed together in a mortar or combined by gentle heat in a beaker or porcelain dish results in a fluid of oily consistence similar in physical characteristics to the well-known campho-phénique. It is without escharotic properties, does not coagulate the cuticle when applied topically, and has high germicidal efficiency. The peculiar value of thymol in the treatment of exposed pulps, even when superficially infected, is well known, and has been the subject of scientific study as well as of practical observation for several years. Solutions of thymol have not heretofore been of much utility in dental practice, owing to the lack of a suitable menstruum; hence the virtues of thymol as a root-canal dressing have not been as practically available as they should be. The combinaton here recommended, and which the writer has taken the liberty of designating as thymophen, is an ideal medicament for use as a canal-dressing, taking the place of campho-phénique or the essential oils, because of its diffusibility, its sedative, non-irritant, and superior germicidal properties. A large clinical use of the preparation is the writer's warrant for strongly recommending it to the dental profession.—Dr. Edward C. Kirk, *Cosmos.*

Restoring Broken Crown Facings.—Grind or clip the pins of the late facing flush with backing. With a lubricated drill make holes through the backing to suit the pins of the new facing. This preparation guides the facing to place, which will be useful during the grinding required to fit the backing. Use thin carbon paper for the fine adjustment of the facing. Bend the ends of the pins to form loops and invest the facing preparatory to uniting the pins with gold or silver solder. While the investment dries and heats, finish preparation of the backing. A U--shaped slot is cut with a small dental fissure-bur, starting in the pinholes already made, and thus making a tongue around which the loop of the facing will fit. A pear-shaped or round bur not too sharp, will cut away enough of the tongue from the palatal side to allow the loop to go to place. Lubricate the bur with vaselin or glycerin.

Having soldered the pins of the facing it will be cool enough now to try it in place for fine adjustment. Cement the facing to place, holding a piece unvulcanized rubber against the palatal aperture to prevent the cement from being forced through the opening. A small ball of soft amalgam at hand quickly closes the aperture in the backing and keeps the loop immovable. The advantages of the method are security of facing, accurate adjustment, and an unweakened dowel in the crown.—W. G. L. Spaulding, *Dominion Dental Journal.*

Erosion.—We usually find erosion existing in the following class of patients: Those who make excessive use of carbohydrates, the gouty, the rheumatic, the anaemic, the tubercular, the scorbutic, and people who usually live very high without dental hygiene. It is, however, more frequent, in the anaemic and neurasthenic class. It is usually accompanied with hyperacidity of the stomach, a fetid acid odor of the breath, and with repeated and frequent eructations. I wish to call attention here to the point that I have never seen cases of erosion existing in the mouths of tobacco-users. It may exist, but I have never observed a case. (It seems to me tobacco may be a good antitoxin for erosion.)—Dr. W. G. Hamm, *Summary.*

Bryant Method of Replacing a Facing.—Have you ever replaced broken facing by Bryant's method? Not the gold nuts, but by connecting the two pins with tinner's solder, filing them into a square shape, cutting a corresponding square box shape hole in gold, backing and cementing into position. An improvement over his way is to grind facing to fit; make an oblong box open at each end, out of steel matrix metal; place over pins, set tooth on stove, pin side up, with box in position, and a piece of Richmond metal on top. A few moments of slight heat and your metal flows into box, around pins, and you have your tooth ready to file to fit the slot in backing; cement into place, and, with warm burnisher melt the metal on lingual surface, smoothing down all the inequalities, and the result is what might be termed a riveted retention. Try it.—Dr. J. P. Root, *Kansa Dental Journal.*

Fees.—A dentist—some dentists—will work and fret and fume and take the foul breath of a patient in the careful, skillful treatment of a putrescent pulp, subdue the inflammation, give relief to the patient, devote, it may be, several sittings to bring the tooth to completion and then charge the paltry sum of $1.50 or $2, or it may be $3, when in fact no one but a dentist could do the work and the patient would not begrudge $5. Again, these dentists who place so low an estimate on the value of their work, will extract a lot of roots and teeth, soil their instruments, spittoon, napkins, and it may be chair and floor, undergo the sickening administration of chloroform or ether, take all the responsibility of accident or death, then take an impression, make a model, secure the bite and make a place for the insignificant sum of $5, or $8, when $10 should be the lowest price and $15 for the better grades of vulcanite work. If we would all work together in these things we would find it to our interest.—C. W. Huntington,*Stom'*

